# In Vivo Estimation of Ketogenesis Using Metabolic Flux Analysis—Technical Aspects and Model Interpretation

**DOI:** 10.3390/metabo11050279

**Published:** 2021-04-28

**Authors:** Stanislaw Deja, Blanka Kucejova, Xiaorong Fu, Jeffrey D. Browning, Jamey D. Young, Shawn Burgess

**Affiliations:** 1 Center for Human Nutrition, The University of Texas Southwestern Medical Center, Dallas, TX 75390, USA; blanka.kucejova@utsouthwestern.edu (B.K.); xiaorong.fu@utsouthwestern.edu (X.F.); 2 Department of Biochemistry, The University of Texas Southwestern Medical Center, Dallas, TX 75390, USA; 3 Department of Molecular Genetics, The University of Texas Southwestern Medical Center, Dallas, TX 75390, USA; 4 Department of Clinical Nutrition, The University of Texas Southwestern Medical Center, Dallas, TX 75390, USA; jeffrey.browning@utsouthwestern.edu; 5 Department of Chemical and Biomolecular Engineering, Vanderbilt University, Nashville, TN 37235, USA; j.d.young@vanderbilt.edu; 6 Department of Molecular Physiology and Bio-physics, Vanderbilt University, Nashville, TN 37235, USA; 7 Department of Pharmacology, The University of Texas Southwestern Medical Center, Dallas, TX 75390, USA

**Keywords:** ketogenesis, stable isotope, ^13^C MFA, LC-MS/MS, ^1^H NMR, in vivo, flux, metabolism, liver, metabolomics, BHB, acetoacetate

## Abstract

Ketogenesis occurs in liver mitochondria where acetyl-CoA molecules, derived from lipid oxidation, are condensed into acetoacetate (AcAc) and reduced to β-hydroxybutyrate (BHB). During carbohydrate scarcity, these two ketones are released into circulation at high rates and used as oxidative fuels in peripheral tissues. Despite their physiological relevance and emerging roles in a variety of diseases, endogenous ketone production is rarely measured in vivo using tracer approaches. Accurate determination of this flux requires a two-pool model, simultaneous BHB and AcAc tracers, and special consideration for the stability of the AcAc tracer and analyte. We describe the implementation of a two-pool model using a metabolic flux analysis (MFA) approach that simultaneously regresses liquid chromatography-tandem mass spectrometry (LC-MS/MS) ketone isotopologues and tracer infusion rates. Additionally, ^1^H NMR real-time reaction monitoring was used to evaluate AcAc tracer and analyte stability during infusion and sample analysis, which were critical for accurate flux calculations. The approach quantifies AcAc and BHB pool sizes and their rates of appearance, disposal, and exchange. Regression analysis provides confidence intervals and detects potential errors in experimental data. Complications for the physiological interpretation of individual ketone fluxes are discussed.

## 1. Introduction

When carbohydrates are scarce, mammalian metabolism resorts to the use of ketones as a compensatory energy source [1]. Ketogenesis occurs in liver mitochondria where long-chain fatty acids (LCFAs) are oxidized to 2-carbon acetyl-CoA units and then condensed into the 4-carbon acetoacetate (AcAc). AcAc, a β-ketoacid, is chemically unstable and is, in large part, reduced to its stable β-hydroxy form, β-hydroxybutyrate (BHB). Hepatic ketogenesis allows the rapid systemic oxidation of lipids by converting insoluble and transport-restricted LCFAs into freely soluble ketones, which are avidly oxidized by most tissues [1,2]. During fasting or carbohydrate restriction, ketone production increases by an order of magnitude and plasma ketone concentrations can rise from 100 μM to several mM. By contrast, glucose production and its plasma concentration rarely change by more than 50%. Yet, unlike endogenous glucose production, which is routinely quantified using stable isotope tracers [3], endogenous ketone production is rarely measured using tracer approaches.

Though less studied than glucose metabolism, ketone metabolism also plays a vital role in the pathology of diabetes, neurological disorders, cardiovascular disease, liver disease, and the inflammatory response [4]. It has been noted that diabetes might have been classified as a disease of fat metabolism, rather than glucose metabolism, if early investigators had first noticed the floral odor of ketosis rather than the glucosuria of their subjects [5]. While hyperglycemia can lead to chronic complications, overactivation of hepatic fat oxidation and ketogenesis can cause rapid death by ketoacidosis in type 1 diabetes [6]. In contrast, obesity and insulin resistance, early factors in type 2 diabetes [7], suppress the ketogenic response [8]. Loss of ketogenesis alone can cause hepatic lipid accumulation, increase oxidative stress, and lead to liver damage [9,10]. A rise in circulating ketones has also been identified as a necessary metabolic adaptation for the immune response during infection [11], and ketones themselves may have anti-inflammatory properties by acting on immune cells [12]. Diets that promote ketosis, such as intermittent fasting [13] and carbohydrate restriction [14], promote weight-loss [15], rapidly improve non-alcoholic fatty liver disease (NAFLD) [16], and are also used to treat neurological disorders [17,18]. Despite the emerging role of ketone metabolism as an important aspect in the molecular physiology of health and disease, quantitative approaches to evaluate the ketogenic pathway in vivo have been difficult to develop, evaluate, and apply.

**Figure 1:**
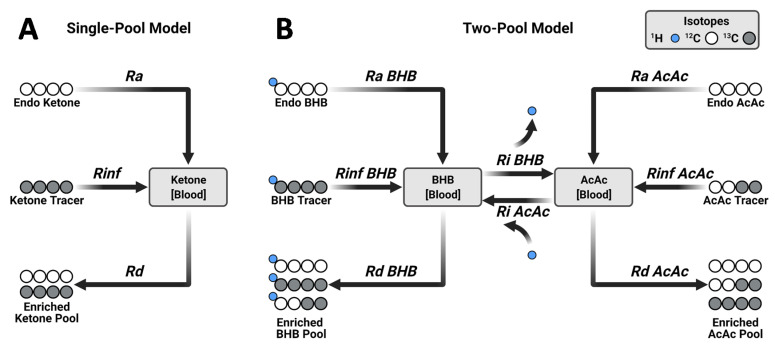
Probing ketogenesis using stable isotope tracers and metabolic flux analysis. (**A**) Single-tracer dilution method for ketone turnover. (**B**) Double-tracer method. Unlike a single-tracer, the double-tracer approach accounts for exchange between BHB and AcAc pools. *Ra*—rate of appearance, *Rinf*—rate of tracer infusion, *Rd*—rate of disposal, *Ri*—rate of interconversion. Panel (**A**) represents the utilization of a uniformly labeled ketone tracer (i.e., [U-^13^C_4_]), while panel (**B**) corresponds to the simultaneous utilization of [U-^13^C_4_]BHB and [3,4-^13^C_2_]AcAc as tracers.

The steady-state production and consumption (i.e., turnover), of a circulating metabolite is typically determined using tracer dilution methods [3]. An isotopically labeled metabolite infused at a known rate is diluted by the endogenously produced, unlabeled metabolite (Figure 1A). At steady-state, the known infusion rate and measured tracer dilution are used to quantify the endogenous synthesis rate [3]. While relatively straightforward for endogenous glucose production, the method is complicated when multiple metabolites are in rapid exchange [19]. For example, the use of a single BHB tracer to measure ketone turnover treats BHB and AcAc as indistinguishable pools [20]. This approach was challenged on theoretical [19,21] and experimental grounds [22] because there is a clear isotope disequilibrium between AcAc and BHB [21]. The biochemical interconversion of these two ketones in peripheral tissue is too rapid to be considered independent, but too slow to be considered indistinguishable [21]. A dual-tracer technique that uses distinguishable AcAc and BHB tracers allowed both the dilution and the interconversion of each ketone to be determined [22,23,24,25,26] (Figure 1B). This approach substantially improved the accuracy of ketone turnover measurements compared to single-tracer approaches and closely matched net hepatic ketone production measured by an arterio-venous (A-V) balance technique [27]. The dual-tracer approach has been used to investigate ketogenesis in preclinical models of obesity [28,29], diabetes [8], and in humans with NAFLD [9]. However, the double-tracer method requires that infusion and enrichment data be fit to a significantly more complex two-pool model.

The standard two-pool model with two endogenous rates of appearance (*Ra*), two rates of disposal (*Rd*), and two rates of interconversion (*Ri*) requires solving six simultaneous equations [24,26]. Matrix approaches easily solve these equations from the two known rates of infusion (*Rinf*) and the measured dilution of the four tracer labeled species [26] (Figure 1B). However, the model assumes fidelity between tracer infusion rates and their dilution by endogenous ketone production and interconversion. Non-ideal processes, such as tracer decomposition, will produce tracer enrichments that can be solved by the matrix model but produce inaccurate, if not physiologically implausible solutions with an unknown certainty.

In the current study, we apply LC-MS/MS detection of ketone enrichment and metabolic flux analysis (MFA) to study ketosis in vivo. We carefully investigate the AcAc tracer stability and plasma AcAc conversion to [^2^H]BHB using ^1^H NMR real-time reaction monitoring. We report that precise determination of the AcAc tracer concentration in the final infusate is critical for accurate flux calculations. We develop a novel implementation of the two-pool model using an MFA approach that regresses all six LC-MS/MS derived ketone isotopologues and two tracer infusion rates simultaneously. This approach provides confidence limits to rigorously evaluate model solutions and can detect data inconsistencies. Finally, using this approach we determine the relationships between individual ketone metabolic fluxes and their respective pools and provide evidence for increasing interconversion of AcAc to BHB as ketosis progresses.

## 2. Results

### 2.1. Tracer Approaches to Study Ketogenesis

**Figure 2:**
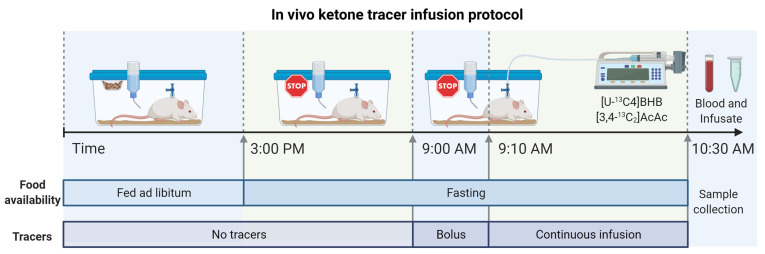
In vivo double ketone tracer infusion protocol in fasted mice. Tracers were administered as a 10-min bolus and 80-min continuous infusion to rapidly achieve and maintain steady-state enrichment. At the conclusion of the experiment, blood and the remaining infusate samples were collected for analysis.

**Figure 3:**
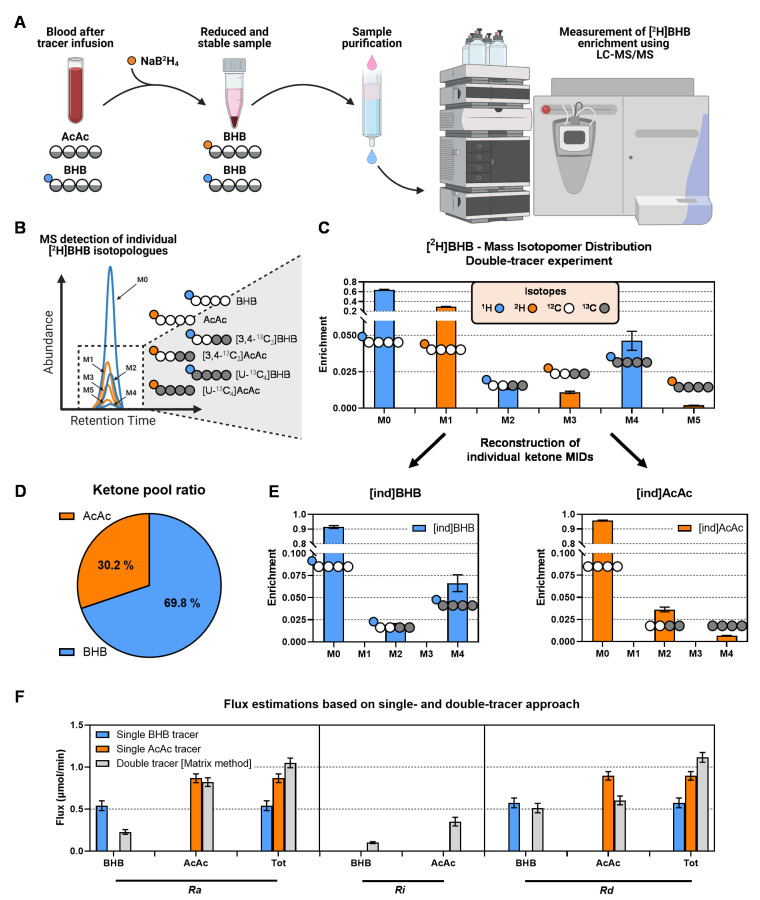
Measurement of ketone enrichment using LC-MS/MS and flux analysis using single- and double-tracer approaches. (**A**) In situ reduction of AcAc by NaB^2^H_4_ forms deuterium-labeled BHB, which preserves unstable AcAc isotopologues as BHB isotopologues with one additional mass unit. (**B**) The combined BHB can be detected using LC-MS/MS and contains isotopologues from BHB (not shifted) and AcAc (shifted by M+1). (**C**) [^2^H]BHB MID—all even isotopologues of BHB after sample preparation are derived from plasma BHB, while all odd isotopologues are derived from plasma AcAc. The MID was corrected for natural abundance using theoretical distributions. (**D**) Odd and even BHB isotopologues encode additional information about relative pool sizes of AcAc and BHB. (**E**) The individual labeling of BHB ([ind]BHB) and AcAc ([ind]AcAc) can be recreated from the odd and even BHB isotopologues of the analyte. (**F**) Comparison of ketone flux determined by using the information from individual BHB and AcAc tracers or both simultaneously (using matrix method). Fluxes were estimated in 14-week-old mice that were fasted for 18-h.

Historically, ketogenesis has been measured using single- (Figure 1A) and double-tracer approaches (Figure 1B) [23,27,30]. To evaluate these methods, we performed double-tracer infusions in 14-week-old mice that were fasted 18-h (Figure 2). A mixture of [U-^13^C_4_]BHB and [3,4-^13^C_2_]AcAc was expected to provide unambiguous labeling of plasma ketones (Figure 1B). To capture AcAc, which can be unstable under some conditions, blood samples were immediately treated with sodium borodeuteride (NaB^2^H_4_) to reduce AcAc to its stable BHB analog with an M+1 mass shift [31] (Figure 3A). Hence, both AcAc and BHB labeling patterns were encoded in the combined [^2^H]BHB mass isotopomer distribution (MID), but any BHB detected as an odd mass (M+1, M+3, M+5) must have originated from an AcAc [29] (Figure 3A,B). Direct inspection of the [^2^H]BHB MID provided evidence of substantial exchange between the two ketones (Figure 3C). We observed significant M+2 BHB which could only come from exchange with the [3,4-^13^C_2_]AcAc tracer in vivo and, to a lesser extent, M+5 BHB which could have only come from the in vivo conversion of [U-^13^C_4_]BHB to AcAc (Figure 3B,C). Importantly, the [^2^H]BHB MID encoded additional metabolic information about the relative pool sizes of AcAc and BHB (Figure 3D).

To assess the limitations of single ketone tracers, we extracted individual MIDs of BHB ([ind]BHB) and AcAc ([ind]AcAc) from the combined [^2^H]BHB MID (Figure 3E) and evaluated their respective ketone body turnovers using a simple tracer dilution analysis (Figure 1A). Next, we compared these results to flux estimates obtained by using both [ind]BHB and [ind]AcAc simultaneously in the matrix implementation of the two-pool model (Figure 3F). Data from the single BHB tracer overestimated *Ra BHB* but provided a good estimate of *Rd BHB* when compared to fluxes obtained using the double-tracer method (Figure 3F). In contrast, the single AcAc tracer provided an accurate *Ra AcAc* while significantly overestimating *Rd AcAc* compared to the two-pool model (Figure 3F). Neither single-tracer approach was able to accurately determine total ketone *Ra* or *Rd*, but the single AcAc tracer performed better overall in capturing total ketone turnover than the single BHB tracer. These errors occur because the single-tracer approaches cannot account for interconversion of the two ketones and are therefore inherently less accurate.

**Figure 4:**
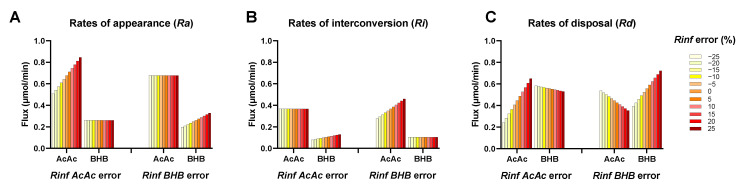
Effect of erroneous infusion rates on (**A**) rate of appearance, (**B**) rate of interconversion, and (**C**) rate of disposal estimated using the matrix solution of the two-pool ketone model (matrix method). The model is prone to errors since it assumes fidelity between tracer infusion rates and their dilution by endogenous ketone production and interchange.

Although the two-pool model is superior compared to the single-tracer approaches, it is not without limitations. In particular, the two-pool model remains vulnerable to systematic errors in infusion rates leading to perturbed mass balances that can alter flux calculations. Since the model uses six constraints (four enrichments and two infusion rates) to solve six fluxes, the solution is uniquely determined (has zero degrees of freedom). The lack of redundancy is not ideal for detecting data inconsistencies or rigorously assessing uncertainties generated by the propagation of errors. To examine the impact of these limitations, we introduced error into one of the tracer infusion rates used to solve the two-pool model. The simulated error had a linear effect on flux estimates (Figure 4A–C). A realistic range of infusion error (+/− 25%) moderately affected *Ra* (Figure 4A) and *Ri* (Figure 4B), while *Rd* was altered by an order of magnitude (Figure 4C), and in some cases negative flux values were obtained (data not shown). Therefore, it is critical to use accurate infusion rates for both tracers, as the model is unable to detect conflicts between the amount of infused material and ketone enrichments. Similarly, loss of higher isotopologues of [^2^H]BHB would result in significant errors in flux estimates (data not shown). Therefore, we moved to validate the technical aspects of AcAc conversion to BHB and the stability of the AcAc tracer.

### 2.2. In Situ Reduction of AcAc to [^2^H]BHB Is Rapid and Complete

AcAc is less abundant than BHB in plasma under most physiological conditions. However, AcAc turnover is rapid, which results in a substantial tracer dilution leading to low AcAc enrichments under many experimental scenarios (Figure 3E). Although a complete AcAc conversion to [^2^H]BHB is not required for flux estimates using the steady-state two-pool model (equations rely only on enrichments, not on absolute ketone ratios), it is important to maintain a reliable level of AcAc for detection of low-abundance isotopologues. Moreover, BHB versus [^2^H]BHB encodes the ratio between AcAc and BHB pools (Figure 3D), which is an important measure of mitochondrial redox, and it may provide additional value in related kinetic or non-steady-state models. Therefore, we evaluated the yield and rate of the in situ reduction of AcAc to [^2^H]BHB.

**Figure 5:**
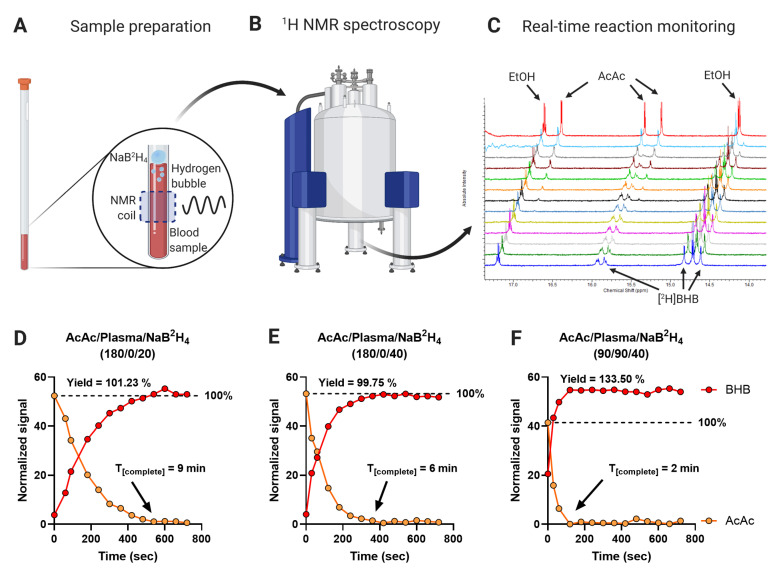
Evaluation of AcAc conversion to [^2^H]BHB by real-time reaction monitoring. (**A**) Sample preparation—reaction of NaB^2^H_4_ with water caused the release of hydrogen resulting in bubbles and poor magnetic field homogeneity. This was prevented by the addition of NaB^2^H_4_ to the surface of the reaction solution, allowing acquisition of NMR spectra. (**B**) The sample was immediately loaded into the NMR spectrometer and (**C**) ^1^H NMR spectra were recorded every 60 s to capture the evolution of BHB resonances over time. (**D**–**F**) Integrated and normalized BHB and AcAc signals over time. A standard sample of 180 µL of AcAc solution was mixed with either (**D**) 20 µL of NaB^2^H_4_ or (**E**) 40 µL of NaB^2^H_4_. (**F**) 90 µL of a mouse plasma sample was mixed with 90 µL of a AcAc solution and topped with 40 µL of NaB^2^H_4_. The concentrations of individual solutions are described in the Materials and Methods section. Signals were normalized to total signal area.

The presence of NaB^2^H_4_ in the solution prevents the use of enzymatic assays for ketone quantification. Therefore, we used ^1^H NMR real-time reaction monitoring to evaluate the conversion of AcAc to BHB. The oxidation of NaB^2^H_4_ generated bubbles in the solution, which disrupted magnetic field homogeneity and signal line shapes. This problem was overcome by adding the reducing reagent to the top of the AcAc solution without mixing (Figure 5A,B). Proton spectra were acquired every 60 s over 12 min (Figure 5C). Despite the lack of mixing during reaction monitoring, 20 μL of NaB^2^H_4_ was sufficient to quantitatively reduce a 180 μL standard solution of AcAc (at higher than physiological concentration) to [^2^H]BHB within 9 min (Figure 5D). This time was decreased to 6 min when 40 μL of NaB^2^H_4_ was used (Figure 5E). To confirm that the reaction was complete in plasma, which contains many other metabolites and proteins that oxidized NaB^2^H_4_, we monitored the reduction of 90 μL of AcAc standard spiked into 90 μL of plasma by 40 μL of NaB^2^H_4_ (Figure 5F). AcAc was quantitatively converted to BHB within 2 min, consistent with the kinetics of the standard solution, indicating that the plasma matrix did not substantially inhibit the reaction. Yields reached 100% in standard solutions (yields: 101.23%, 99.75%) and exceeded 100% (133.50%) in the plasma sample, likely due to more challenging line shapes and the potential contribution from metabolites that co-resonate with BHB in the plasma sample. These data indicate that the conversion of AcAc to [^2^H]BHB is rapid, complete, and unaffected by the presence of other metabolites and proteins in the sample. Inasmuch as the LC-MS/MS sample preparation protocol used a greater proportion of NaB^2^H_4_ per sample (35 μL of reagent and 25 μL of sample) than the NMR real-time reaction monitoring experiments, we conclude that the conversion of AcAc to [^2^H]BHB provides an accurate determination of the AcAc and BHB pools.

### 2.3. Stability of the AcAc Tracer

**Figure 6:**
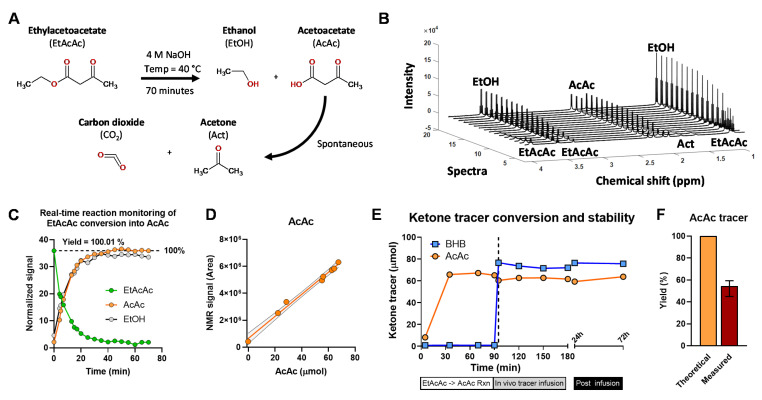
Conversion of EtAcAc to AcAc and stability of AcAc tracer. (**A**) Hydrolysis of EtAcAc to AcAc and EtOH (**B**) Real-time reaction monitoring by ^1^H NMR. (**C**) Integrated ^1^H NMR resonances of EtAcAc, AcAc, and EtOH over time. (**D**) The relationship between AcAc detected using ^1^H NMR and an enzymatic assay. (**E**) Concentration of BHB and AcAc tracers during infusate preparation, double-tracer in vivo infusion in mice, and post-infusion. Dashed line denotes mixing of AcAc and BHB tracer solutions. (**F**) Measured AcAc tracer yield, data presented as mean and range.

Acetoacetate is a β-ketoacid and, like other members of this class, is unstable [32]. The acidic form of AcAc has a half-life of less than 3 h and undergoes spontaneous decarboxylation to acetone and CO_2_, though the basic form may be stable for days in solution [32]. The ethyl ester of AcAc (EtAcAc) is commonly used as a stable source of AcAc but must be hydrolyzed at high pH and neutralized immediately before infusion (Figure 6A). Thus, the decomposition of AcAc tracer is possible during the pH adjustment procedure. Loss of AcAc tracer could lead to lower-than-expected infusion rates which would significantly affect the flux estimates (Figure 4).

To examine these possibilities, we again used the real-time reaction monitoring approach. We utilized ^1^H NMR to evaluate the hydrolysis of EtAcAc to AcAc under base catalyzed conditions (Figure 6B and Figure S1A–D). The reaction was ~75% complete within 15 min and quantitative after 70 min (Figure 6C). While acetone is the major product of AcAc decomposition, it consisted far less than 1% of the AcAc signal, suggesting that spontaneous decarboxylation was not a significant source of AcAc loss during the reaction conducted under basic conditions (Figure S1D,E). Samples from the reaction solution demonstrated excellent correlation between the AcAc concentration measured by enzymatic assay and the integrated ^1^H NMR signal of AcAc (Figure 6D). Likewise, there was an excellent inverse correlation between the EtAcAc NMR signal and AcAc concentration (Figure S1F). Next, we quantified AcAc concentration throughout the duration of a tracer infusion experiment and found no decrease in AcAc content during this time-period (Figure 6E). When the infusate was kept in the syringe pump at room temperature there was no significant change in the AcAc or BHB content after 24- and 72-h post-infusion (Figure 6E and Figure S1G).

Nonetheless, there was a ~40% lower measured concentration of AcAc in the infusate compared to the theoretical amount expected from the weight of EtAcAc used (Figure 6F), while BHB was closer to its expected concentration. The loss of AcAc most likely occurs during neutralization of the hydrolysis reaction with acid solutions, especially if the pH drops below 7. Thus, EtAcAc hydrolysis is quantitative, but infusate AcAc can be lost during pH adjustments and should therefore be measured in the final infusate to determine accurate flux estimates.

### 2.4. MFA Implementation of the Two-Pool Model of Ketogenesis

**Figure 7:**
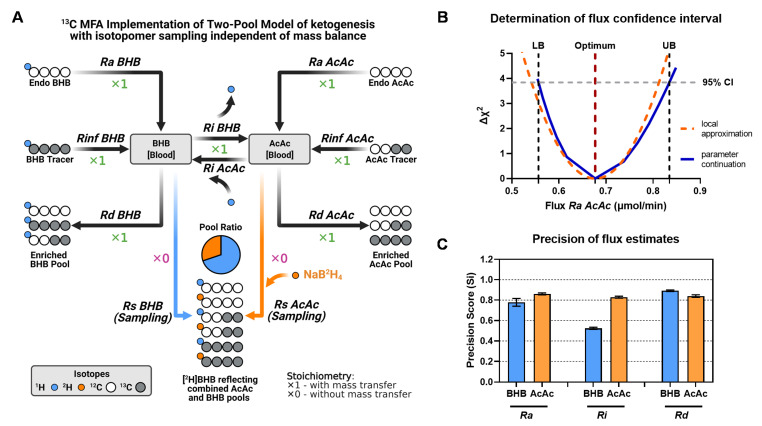
A novel MFA implementation of the two-pool model of ketogenesis. (**A**) Isotopomer model structure. Note that additional sampling reactions (*Rs*) for BHB and AcAc have a stoichiometric coefficient of 0 to access labeling information but prevent the reaction from being part of the mass balance network. Unlike the matrix method, the MFA model encodes the BHB and AcAc pool ratio, although steady-state flux estimates are independent of this information. (**B**) Determination of confidence intervals (CI) for *Ra AcAc*. The MFA version of the two-pool model of ketogenesis allows determination of individual CIs by calculating lower (LB) and upper (UB) boundaries. (**C**) Precision of flux estimates. Precision scores were determined based on the normalized range of confidence intervals across all samples used for the model development. Precision scores were calculated for fluxes estimated in 14-week-old mice that were fasted for 18-h (see Figure 3).

We hypothesized that the MFA model, using an elementary metabolite unit (EMU) framework [33], would provide better rigor for modeling ketogenic fluxes by allowing the determination of confidence intervals. These models can regress all relevant isotopomers and infusion rates by assigning weights to errors in individual measurements and have recently become a standard approach. We designed the MFA model with a structure similar to the two-pool matrix model but allowed it to utilize the combined [^2^H]BHB MID rather than the reconstructed AcAc and BHB isotopologues. This format incorporates the conversion of AcAc into [^2^H]BHB during the sample preparation phase (Figure 7A). The atom transitions used in the network are schematically presented in Figure 7A and reported in detail in Table 1. Note that this model utilizes additional “pseudofluxes” assigned to sampling reactions (*Rs*) for BHB and AcAc. They represent the removal of an infinitely small sample from the system and, hence, have a stoichiometric coefficient of 0. This function allows isotopomer enrichments of blood BHB and AcAc pools to be modeled without affecting the mass balance of the network. The ratio of AcAc and BHB in plasma is indicated by the sampled [^2^H]BHB isotopomers, and is an adjustable parameter that is estimated during the data fitting procedure, but is independent of steady-state fluxes. Similar to the matrix method, the MFA ketone turnover model (Figure 7A) was characterized by zero degrees of freedom (DOF = 0), and thus resulted in a uniquely determined solution.

We used the MFA model of ketogenesis to examine the experimental data from 18-h fasted mice, previously investigated by the tracer dilution and two-pool model matrix analysis (Figure 1, Figure 2 and Figure 3). Regression of these data resulted in flux estimates identical to the matrix method but also provided confidence intervals for individual fluxes (Figure 7B). We used these data to evaluate the precision of individual flux estimates using a precision score metric (Figure 7C). We found that most ketogenic fluxes were characterized by high precision scores, with values around 0.8. Only *Ri BHB* was found to be less precise, with a precision score of 0.5.

Finally, unlike the matrix model, MFA based on the two-pool model allows the unbiased detection of data inconsistencies. Both matrix and MFA models are uniquely determined, and therefore vulnerable to experimental errors. While a matrix solution may solve a flawed data set to physiologically implausible flux values (e.g., negative fluxes), MFA constrains solutions to physiological ranges and, in such cases, would result in a mismatch between the experimental data and modeled data. By enforcing mass balance boundaries, MFA also identifies the MID data that would have driven the model out of range, providing context to data that can be objectively excluded from the analysis (Figure S2A,B).

### 2.5. Ketone Fluxes Change Non-Linearly with the Progression of Ketosis

**Figure 8:**
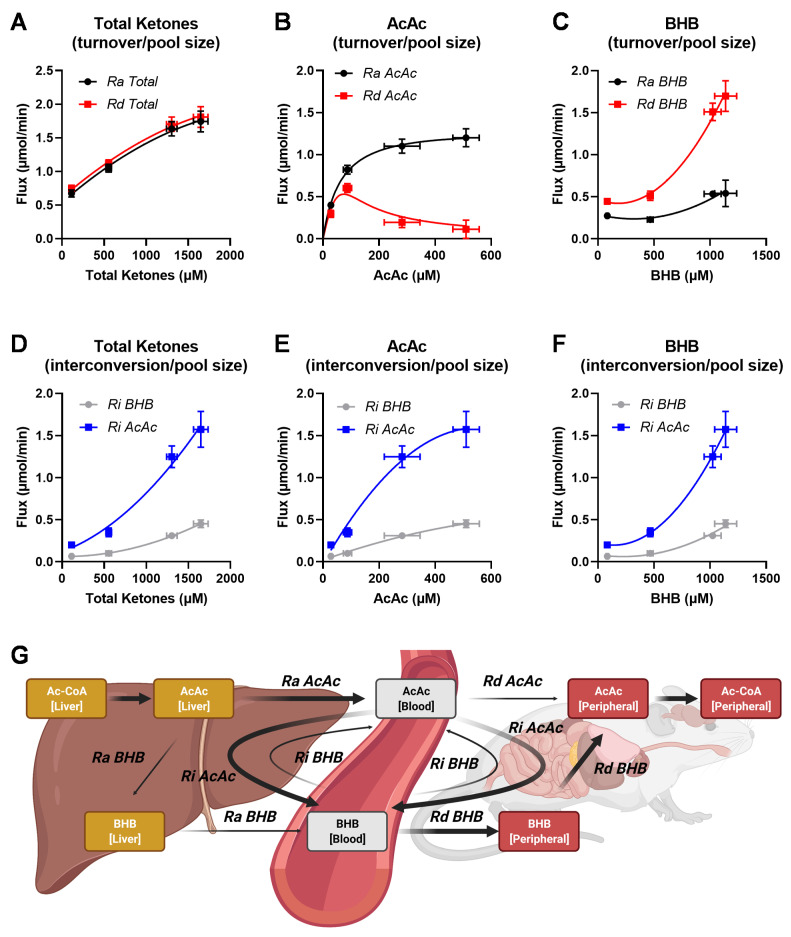
The relationship between fluxes and individual ketone pool sizes. Ketone turnover in relationship to pool size: (**A**) total ketones, (**B**) AcAc, and (**C**) BHB. Relationships between interconversion flux and the individual plasma pool sizes for (**D**) total ketones, (**E**) AcAc, and (**F**) BHB. *Ra*—rate of appearance, *Rd*—rate of disposal, *Ri*—rate of interconversion. (**G**) Schematic representation of ketogenic flux in the whole body. The MFA model suggests that ketones are primarily generated in the form of AcAc. As ketosis progresses, changes in the BHB pool and BHB disposal are driven by the interconversion of AcAc to BHB in various tissues. At high plasma ketone concentrations, the majority of ketone disposal occurs through the BHB pool. Complications for interpretation of these results are presented in the Discussion section. Fluxes were estimated in 14-week and 20-week-old mice that were either fed ad libitum or fasted for 18-h. Groups appear on the graphs from left to right in the following order: 20-week-old fed ad libitum, 14-week-old 18-h fasted, 20-week-old 18-h fasted, and obese (HFD) 20-week-old 18-h fasted.

We used the MFA model to estimate ketogenic fluxes under different physiological conditions that should either stimulate or attenuate ketogenesis. We conducted three additional infusion experiments in 20-week-old mice: fed ad libitum, fasted 18 h, and fasted 18 h obese mice (fed a high fat diet (HFD)). Together with our initial dataset obtained in 14-week-old mice fasted for 18 h, we monitored four different states of ketone metabolism. Interestingly, younger mice had lower plasma ketone concentrations than older mice (Figure S3A). We suspect that lower ketogenesis was due to the lower body weight of younger mice (Figure S3B), which may have been limiting during an extended fasting duration (18-hrs). Together, these groups provided a wide range of physiological ketosis, without reaching the pathological levels observed during ketoacidosis (Figure S3A). To better understand the relationship between individual fluxes and their respective ketone pools, we additionally measured individual BHB and AcAc concentrations in a subset of samples, which allowed analysis of metabolite-flux relationships (Figure 8).

As the total ketone *Ra* and *Rd* rose, we observed an increase in plasma total ketone concentration (Figure 8A). This relationship was curvilinear, as the flux values gradually plateaued despite increasing pool sizes (Figure 8A). The transition from fed to fasted state caused a massive 12- to 17-fold increase in total plasma ketones, while total ketone turnover increased only by 2.5-fold. Interestingly, this relationship varied between the individual ketone fluxes. An increase in plasma AcAc concentration was associated with increased *Ra AcAc* initially, but it plateaued at concentrations above 300 μmol/L. *Rd AcAc* initially increased with concentration, similar to *Ra AcAc,* but it decreased at higher concentrations (Figure 8B). In contrast to AcAc, *Ra BHB* remained relatively unchanged across a wide range of plasma BHB concentrations, while *Rd BHB* rose rapidly with an increase in plasma ketone concentration (Figure 8C). These data indicate a divergence between individual ketone *Ra* and *Rd* as the total pool of ketones increases.

Ketone interconversion rates also showed distinctive associations with individual ketone pool sizes. Although both *Ri AcAc* and *Ri BHB* increased with the total ketone plasma concentrations, the *Ri AcAc* was consistently higher than *Ri BHB* (Figure 8D). Importantly, *Ri AcAc* started to plateau with increasing concentrations of plasma AcAc (Figure 8E), yet it increased rapidly as the BHB concentration exceeded 500 μmol/L values (Figure 8F). Since *Ri AcAc* generates BHB from AcAc, these data suggest that the rise in plasma BHB concentration occurs primarily due to more active *Ri AcAc* flux. On the other hand, *Ri BHB* also increased with rising plasma ketones but not to the same extent as *Ri AcAc*. As a result, the discrepancies between the two interconversion rates escalated as ketosis progressed. Therefore, as ketosis develops, the blood BHB pool increases due to secondary interconversion of AcAc to BHB, rather than the increased direct de novo release of BHB. This exchange might occur in the liver or other peripheral tissues, but the model detects that a substantial portion of the new AcAc mixes with the blood pool before conversion to BHB (Figure 8G).

## 3. Discussion

The multiple and exchanging ketones of the ketogenic pathway require a dual-tracer approach to be quantified. Although the dual-tracer approach was developed more than 3 decades ago, application of a single [U-^13^C_4_]BHB tracer is still fairly common [34,35]. Single tracers are able to detect general changes in flux in response to physiological changes in ketosis, but they do not account for the ketone interconversions and may therefore lack accuracy. Recent interest in measuring ketogenesis has been stimulated by emerging evidence for the role of ketones in a variety of diseases, and its stimulation by pharmacological and dietary interventions. Thus, we validated the technical challenges associated with the dual-tracer method and developed a novel approach for implementing the two-pool model using a modern MFA approach.

### 3.1. Technical Aspects of In Vivo Estimation of Ketogenesis

The dual-tracer approach is significantly more complex than a single-tracer dilution experiment and has numerous additional technical challenges. First, two additional steps are required to overcome the instability of AcAc as an analyte and tracer. Decomposition of AcAc to acetone occurs naturally in circulation, and plasma acetone has been reported at near mM concentrations in 3-day fasted humans [36]. It is unclear how much acetone formation contributes to AcAc disposal, but in humans it was a maximum of ~30% after 21-days of starvation [36]. Nevertheless, in vivo acetone formation should not affect model mass balances since primary and secondary tracers of AcAc would equally decompose, and therefore would be indistinguishable from conventional *Rd AcAc*. In contrast, decomposition of AcAc during analysis would give an underestimate of plasma AcAc concentration. Treatment of samples with NaB^2^H_4_ allows labile AcAc to be preserved as a ^2^H-tagged BHB for analysis [31,37] (Figure 3A). However, to the extent that the reaction is incomplete, there would be a selective loss of all AcAc isotopomers relative to BHB isotopomers. Fortunately, the structure of the two-pool model is independent of yield since the mass balance equations rely only on fractional enrichments of the mass isotopomers and not the MIDs of AcAc and BHB relative to each other. Nevertheless, other implementations might benefit from evaluating the relative [BHB]:[AcAc] pool ratio and isotopomers. We show that AcAc conversion to [^2^H]BHB is rapid and complete. These data are in agreement with rapid conversion rates observed for other ketoacids using this method [38] and confirms that incomplete conversion need not contribute to discrepancies in AcAc quantification [31]. Therefore, in situ reduction of AcAc to BHB prevents loss of labeling data encoded in AcAc and, since it provides quantitative yields, the method also preserves the ketone ratio which may provide insight into mitochondrial redox. However, reaction conditions may vary among laboratories or applications, so it is recommended that this assumption be evaluated on a case-by-case basis if information regarding relative [BHB]:[AcAc] pool ratio is desired.

Secondly, most AcAc salts are unstable, so AcAc tracers are sold in the ester form, typically ethylacetoacetate (EtAcAc). EtAcAc is hydrolyzed in a basic aqueous solution and neutralized just prior to infusion. Loss of AcAc tracer could occur due to poor yields or the decomposition of AcAc during the infusion experiment, with either causing a mass imbalance that would lead to inaccurate flux estimates. Although yields for the hydrolysis of EtAcAc were near quantitative, a significant loss occurred during neutralization with acid. However, once prepared at neutral pH, the AcAc tracer remained stable throughout the infusion protocol. Therefore, it is critical to measure the actual concentration of AcAc in the infusate samples following infusion, as it may not match the theoretical yield of the EtAcAc used to prepare the solution.

### 3.2. Advantages of the MFA Implementation of a Two-Pool Model of Ketogenesis

Metabolic flux approaches based on EMU frameworks provide a standardized form of metabolic model notation and concise regression procedures [33]. This approach can be tailored to complex networks without exhaustive mass balances on all possible isotopomers and are thus ideal for in vivo flux models which combine multiple and overlapping metabolic networks [39,40,41,42,43]. For example, recently we developed a unified positional and mass isotopomer model of the TCA cycle and gluconeogenesis, which allows analysis of MS isotopologues and/or NMR isotopomers [42]. Likewise, the MFA model of ketogenesis can be easily modified to accommodate ^13^C NMR ketone measurements [8]. More recently, these models were modified to account for Cori cycling and incomplete exchange reactions [43]. An important point is that comprehensive models can be built over time by adding new networks, analytes, or types of isotopomer information without recoding mass equations for every possible isotopomer. By developing the two-pool model of ketogenesis in an EMU framework, we intend to allow its unification with clearly related networks, such as the hepatic TCA cycle, which have already been employed [42].

Like previous approaches, the MFA method uses a two-pool non-compartmental model with interconverting AcAc and BHB. Previous methods rely on isotopomer ratios and mass balance equations that provide exact solutions, but are rarely regressed to quantify confidence intervals or provide goodness-of-fit metrics. Importantly, small differences in isotopomer ratios can have a large impact on flux estimates, including providing physiologically implausible (negative) fluxes. The nature of these errors is challenging to identify by visual inspection of the measurements or even the flux estimates obtained using the matrix method. The MFA approach, which allows weights of individual measurements to be assigned and regresses all variables into a single metabolic model, was able to indicate the nature of data inconsistencies by identifying which experimental data were poorly fit to the modeled solution. While the matrix equations may provide physiologically implausible solutions under these conditions, the MFA model reports simulated data and allows evaluation of the measurements, in addition to flux values. We also applied the precision score developed by Metallo et al. [44] to well-fit data, which allowed the determination of individual flux precision values. These advantages provide the ability to detect inherent errors in a tracer experiment, such as analytical errors, which result in the unacceptable regression of data.

The two-pool MFA model of ketogenesis evaluates a single BHB MID and thus can extract the BHB to AcAc ([^2^H]BHB) pool ratio. Unfortunately, this information does not provide additional degrees of freedom to the model, since steady-state flux estimates are independent of pool size. However, this information may be beneficial for non-steady-state or dynamic conditions where the ketone pool ratio would provide additional modeling constraints.

### 3.3. Interpretation of Ketone Interconversions in Fasted Mice

The two-pool model accounts for the interconversion of BHB and AcAc but does not distinguish between different tissue compartments. This model mixes hepatic and peripheral interconversions into a single network represented by plasma ketone isotopomers (Figure 8G), which presents some ambiguities regarding the nature of these fluxes. *Ra BHB* and *Ra AcAc* are presumed to occur in liver, since no other tissue is capable of significant rates of net ketone production [4]. *Rd*s are presumed to occur in peripheral tissues, since liver lacks succinyl-CoA:3-ketoacid CoA transferase (SCOT), an enzyme necessary for ketone oxidation [4]. While *Ra BHB* naturally includes the conversion of AcAc to BHB in liver, it is not considered a component of *Ri,* but rather new BHB that traversed the AcAc pool without mixing with ketone tracers. The extent to which AcAc mixes with tracer AcAc in the liver, before conversion to BHB, is unclear. If intrahepatic mixing were complete, *Ra BHB* would not be detected, and all ketone production would be indicated by *Ra AcAc* with subsequent exchange to BHB. If intrahepatic mixing were non-existent, *Ra AcAc* and *Ra BHB* would quantitatively represent AcAc release and BHB release, respectively. Previous investigations demonstrated the disequilibrium of these pools and, for this reason, most applications of the two-pool model use the sum of *Ra AcAc* and *Ra BHB* to represent total ketone turnover rather than report the individual fluxes [23]. Indeed, we found that new AcAc release exceeded BHB release, which is contrary to their liver and plasma concentrations. The [BHB]:[AcAc] ratio and the equilibrium constant of the BHB dehydrogenase reaction are classically used to examine mitochondrial redox state. Under most conditions the [BHB]:[AcAc] ratio in rodent liver is between 2 to 4, depending on nutritional or hormonal state [45]. Presumably, this ratio should be captured in the relative *Ra*s. However, the *Ra BHB* to *Ra AcAc* ratio was less than 1 in all the conditions we examined. This could indicate that partial mixing of BHB and AcAc tracers occurs in liver, making individual *Ra*s difficult to interpret, though their sum is reflective of total ketone turnover [27]. However, if the transport of AcAc out of liver is faster than BHB, then the [BHB]:[AcAc] ratio could be >1 while the ratio of *Ra BHB* to *Ra AcAc* is still <1.

Nevertheless, these apparent fluxes are affected differentially by conditions that alter ketosis. Data from a range of conditions in mice indicate that ketosis does not occur by inducing proportional changes in BHB and AcAc appearance (Figure 8). Rather, a rise in plasma AcAc concentration was accompanied by an initial curvilinear 3-fold increase in *Ra AcAc*, but a dramatic decline in *Rd AcAc*. Conversely, rising plasma BHB concentrations were associated with a curvilinear 4-fold increase in *Rd BHB* but had no effect on *Ra BHB*. *Ri AcAc* and *Ri BHB* increased with rising concentrations of either total ketones or individual ketones, but *Ri AcAc* tended to be more active than *Ri BHB*. These data suggest that changes in plasma AcAc concentration were primarily driven by alterations in *Ra AcAc*. However, this was not the case for BHB, where increases in the BHB pool size occurred despite little change to *Ra BHB* and a large increase in *Rd BHB*. The rise in BHB in this cohort of animals appeared to be related to increased *Ri AcAc* (i.e., new AcAc converted to BHB) relative to *Ri BHB* (i.e., new BHB converted to AcAc). The ratio of *Ri BHB:Ri AcAc* was 2-4, with higher ratios being related to more ketotic states. This finding is in line with the expected effect of hepatic redox state on the β-hydroxybutyrate dehydrogenase (BHBDH) equilibrium. Hence, it is tempting to posit that *Ri* fluxes reflect hepatic redox by tracking the forward and reverse reactions of BHBDH. However, human muscle was also observed to convert AcAc to BHB and release it back to circulation [46,47,48]. A similar high peripheral interconversion rate of AcAc to BHB was observed in fasted human infants [24], which was suggested to support brain function by providing sufficient BHB during glucose scarcity [24]. The [BHB]:[AcAc] ratio (and apparent redox state) of muscle is comparable to liver in magnitude and response to fasting in humans, suggesting that the exchange of these two metabolites could likewise represent muscle BHBDH activity [49]. Nevertheless, it is worth noting that BHBDH activity in rodent liver is more than an order of magnitude higher than in skeletal muscle and heart [50,51]. Thus, additional tissue-specific experiments are required to determine whether *Ri*, or indeed plasma [BHB]:[AcAc] ratios, are representative of tissue-specific mitochondrial redox state.

Finally, our study had a few limitations. First, the variety of conditions used to observe the relationship between plasma ketone concentrations and fluxes (Figure 8) were not inherently independent or systematic manipulations of ketosis. The four groups, a combination of 14- and 20-weeks old, lean and obese mice in the fed or fasted state, represent a broad selection of ketogenic states studied in many labs. However, factors that affect the ketone fluxes may vary among mouse cohorts. For example, although we observed that BHB accumulation was related to *Ra AcAc* and *Ri AcAc*, this might be different in unrelated models of ketosis, such as type 1 diabetes. The data are only meant to demonstrate that *Ra*, *Rd,* and *Ri* can combine in a variety of ways to alter the ketone pools. Secondly, although our study focused on technical aspects of quantifying ketone fluxes in vivo, we recognize that only male mice were used and the discovered relationships between ketone pools and individual fluxes might be different in female mice. Moreover, as discussed herein, the nature of the individual fluxes is incompletely understood. In addition to partial equilibration of ketone tracers in liver, another consideration is the dilution of circulating ketone tracers by exchange with peripheral acetoacetyl-CoA, or so called pseudoketogenesis [52,53]. The exchange of labeled acetoacetate and acetoacetyl-CoA (derived from fatty acid oxidation in muscle tissue, for example) in the SCOT reaction is a potential source of overestimation of acetoacetate dilution and has been discussed extensively [23,29,52,53]. The fidelity between splanchnic A/V ketone release and total ketone *Ra* suggests that the process does not alter the two-pool model estimate of total ketone *Ra*, at least in the canine model [27]. We cannot presume that the process does not impact the individual six fluxes of the model, since pseudoketogenesis may specifically dilute the acetoacetate isotopomers relative to BHB isotopomers. In the future, the ability to examine these pools relative to each other in the combined BHB MID of multiple compartments (e.g., liver, blood and muscle) may provide additional degrees of freedom in an appropriate model of ketone kinetics and pseudoketogenesis.

In summary, we describe a novel MFA analysis of a two-pool model of ketone metabolism. The approach regresses all six ketone isotopologues from AcAc and BHB tracers, and their infusion rates, to obtain estimates of *Ra*, *Ri*, *Rd,* and their confidence intervals. Residuals of individual data can alert the user to potential errors in data. Finally, the EMU nature of the model will allow it to be combined with additional data and more sophisticated metabolic networks in the future.

## 4. Materials and Methods

### 4.1. Animals

Animal protocols were approved by the Institutional Animal Care and Use Committee at the University of Texas Southwestern Medical Center (APN: 2018-102548). Male C57BL6/J mice were maintained on a 12-h/12-h dark/light cycle, with unrestricted access to food and water unless otherwise noted. Animals were fed either a normal chow diet (NCD; Teklad Diet 2016; Harlan Laboratories, Indianapolis, IN, USA) or high-fat diet (HFD, 60% fat calories, Teklad Diet TD06414; Harlan Laboratories) for 16 weeks. Experiments were carried out in 14-week and 20-week-old mice that were fasted for 18-h (between 3PM-9AM) in cages with false bottoms to prevent coprophagia.

### 4.2. Stable Isotope Tracer Preparation

All tracer solutions were freshly prepared on the day of infusion, as previously described [29]. Briefly, a solution of approximately 20 mM [U-^13^C_4_]BHB and 30 mM [3,4-^13^C_2_]acetoacetate was prepared. For example, 11.66 mg of [U-^13^C_4_]BHB was dissolved in 1.78 mL of saline (0.9% NaCl) and kept on ice. In a separate vial, 15.56 mg of [3,4-^13^C_2_]ethylacetoacetate was mixed with 2.22 mL of H_2_O and 44.8 μL of 4 M NaOH, and immediately placed in a heatblock at 40 °C for 70 min. The solution was placed on ice and neutralized using 1 M HCl. BHB and AcAc solutions were combined and used for tracer infusions. Final concentrations were determined by enzymatic ketone assay.

### 4.3. In Vivo Double-Tracer Infusions

Mice were surgically implanted with indwelling jugular vein catheters and allowed to recover for 4 days. During that time, body weight was monitored daily and loss never exceeded 10% of body weight prior to surgery. Tracers were administered as a 10-min prime infusion (5.21 and 4.77 μmol/hr of [U-^13^C_4_]BHB and [3,4-^13^C_2_]AcAc, respectively) followed by an 80-min continuous infusion (2.08 and 1.91 μmol/hr, respectively) similar to previous studies [28,29,54]. These infusion rates provided between 1 and 10% plasma enrichments, which were sufficient for analysis but not too high to break tracer assumptions. Consideration of infusion rates should be made to result in measurable plasma ketone enrichments but low enough to maintain tracer status. Animals were allowed unrestrained movement within the cage during the entire infusion period but had no access to chow or water. At the end of the infusion, mice were anesthetized with isofluorane and blood was collected by tail or cheek bleed into tubes containing EDTA. Samples were stored at −80 °C until analysis.

### 4.4. Measurement of Ketone Body Concentration

Ketone concentrations in infusate samples were quantified enzymatically using Total Ketone and BHB autokit (FUJIFILM Wako Diagnostics U.S.A. Corporation, Mountain View, CA, USA). Infusate samples were diluted 30× with H_2_O to match the dynamic range of the method. A 4 µL sample was used for the assay following manufacturer’s instructions.

### 4.5. In Situ Reduction of AcAc and Sample Purification

Blood samples were left on ice to thaw prior to sample preparation. Briefly, 25 μL of blood was transferred into a fresh Eppendorf tube and mixed with 30 μL of freshly prepared 1.8 M sodium borodeuteride (NaB^2^H_4_) in 0.1 N NaOH. Samples were vortexed and left for 5 min at room temperature to complete the reaction [31,38]. Next, 55 μL of acetonitrile was added and samples were centrifuged for 10 min in 4 °C at 21,000× *g*. Solution (including any residual foam) was transferred to a previously prepared Dowex column (50WX8 hydrogen form (100–200 mesh)), while avoiding solid debris. Centrifugations and transfers were repeated to minimize sample loss. Sample was eluted with 3 mL of ultrapure water and collected into 4 mL glass vials. Samples were freeze-dried in a speed vacuum concentrator, yielding a white powder.

### 4.6. Measurement of Ketone Enrichment by LC-MS/MS

Ketone enrichment was determined by LC-MS/MS as described previously [29], although other approaches may also be applicable [55]. Briefly, dried samples were dissolved in 60 μL of MeOH:H_2_O (2:98) with 0.015% acetic acid, vortexed, and transferred into Eppendorf tubes. Samples were centrifuged for 10 min at 4 °C at 21,000× *g* and supernatants were transferred to LC-MS vials before analysis. The analysis was performed using a reverse phase C18 column (Waters Atlantis T3, 150 × 2.1 mm, 3 µm). Mobile phase consisted of water/methanol (98:2, vol/vol) with 0.0125% acetic acid (eluent A) and water/methanol (60:40, vol/vol) with 0.0125% acetic acid (eluent B). The gradient started with 0% B, and increased to 20% B over 10 min, followed by an increase to 90% B over 0.5 min for 4 min, and equilibrating within 7 min. The analytes were detected by ESI-MS/MS using an API 3200 triple-quadrupole LC-MS/MS system equipped with an ESI Turbo Ion Spray interface, operated in the positive ion mode (AB Sciex, Framingham, MA, USA). The ion source parameters were set as follows: curtain gas: 20 psi, ion spray voltage: 3000 V, ion source temperature: 400 °C, and nebulizing and drying gas: 30 and 40 psi. Triple-quadrupole scans were acquired in the multiple reaction monitoring (MRM) mode with Q1 and Q3 set at “unit” resolution. The following MRM transitions were monitored to quantify BHB isotopologues: 105/87 (M+0), 106/88 (M+1), 107/89 (M+2), 108/90 (M+3), 109/91 (M+4), and 110/92 (M+5) with collision energy of 8.1 V and declustering potential of 26 V. Individual isotopologue origins are explained in Figure 1B and Figure 3. Resulting MIDs were corrected for natural abundance.

### 4.7. Real-Time Reaction Monitoring by ^1^H NMR Spectroscopy

^1^H NMR real-time reaction monitoring was used to evaluate dynamics and yields of two different reactions: (1) in situ reduction of AcAc to [^2^H]BHB and (2) hydrolysis of EtAcAc to AcAc. All spectra were recorded using 14.1 T Varian Inova NMR spectrometer (Varian Instruments, Palo Alto, CA, USA) equipped with a 3 mm broadband probe. For AcAc reduction to [^2^H]BHB, a standard infusate sample was prepared containing ~30 mM [3,4-^13^C_2_]AcAc. AcAc solution was placed in 3-mm NMR tubes and topped with 1.8 M NaB^2^H_4_ in 0.1 M NaOH. In one reaction, additional volume of blood plasma was used to account for reaction with other metabolites and proteins. The following proportions were used: (1) 180 μL AcAc with 20 μL NaB^2^H_4_; (2) 180 μL AcAc with 40 μL NaB^2^H_4_; (3) 90 μL AcAc with 90 μL plasma; and 20 μL NaB^2^H_4_. Once combined with reducing reagent, sample was immediately loaded into the NMR spectrometer and ^1^H NMR spectra were recorded every 60 s to capture the evolution of BHB resonances over time. For hydrolysis of EtAcAc to AcAc, sample was prepared according to standard infusate protocol. 200 μL of EtAcAc freshly mixed with 4 M NaOH was placed in a 3-mm NMR tube and loaded into the NMR spectrometer maintained at 40 °C. ^1^H NMR spectra were recorded manually every 3–5 min for 70 min. The same reaction was simultaneously conducted in the heat block using the remaining aliquot of the original infusate preparation and sampled every 10–15 min for concentration assays. All spectra were zero-filled, manually phased, and baseline corrected in ACD/Labs 12.0 software (Advanced Chemistry Development, Inc., Toronto, Canada) and exported to Matlab for signal integration and 3D spectra visualization.

### 4.8. Flux Calculations Using Analytical Equations (Matrix Method)

Equations were adapted from Fletcher et al. [26]. Briefly, when the two-pool model (Figure 1B) is at steady-state it can be represented by a system of 6 linear equations that describe all mass balances in the system.

(1) $$\mathrm{Total}\;\mathrm{AcAc}\;\mathrm{balance}:\;Rinf_{AcAc}+Ra_{AcAc}+Ri_{BHB}=Rd_{AcAc}+Ri_{AcAc}$$

(2) $$\mathrm{Total}\;\mathrm{BHB}\;\mathrm{balance}:\;Rinf_{BHB}+Ra_{BHB}+Ri_{AcAc}=Rd_{BHB}+Ri_{BHB}$$

(3) $$[3,4{-}^{13}C_{2}]\mathrm{AcAc}\;\mathrm{balance}:\;Rinf_{AcAc}+B\times Ri_{BHB}=A\times Rd_{AcAc}+A\times Ri_{AcAc}$$

(4) $$[U{-}^{13}C_{4}]\mathrm{BHB}\;\mathrm{balance}:\;Rinf_{BHB}+C\times Ri_{AcAc}=D\times Rd_{BHB}+D\times Ri_{BHB}$$

(5) $$[3,4{-}^{13}C_{2}]\mathrm{BHB}\;\mathrm{balance}:\;A\times Ri_{AcAc}=B\times Ri_{BHB}+B\times Rd_{BHB}$$

(6) $$[U{-}^{13}C_{4}]\mathrm{AcAc}\;\mathrm{balance}:\;D\times Ri_{BHB}=C\times Ri_{AcAc}+C\times Rd_{AcAc}$$

where *A*, *B*, *C*, and *D* are steady-state enrichments of [3,4-^13^C_2_]AcAc, [3,4-^13^C_2_]BHB, [U-^13^C_4_]AcAc, and [U-^13^C_4_]BHB, respectively. These 6 equations were rearranged into matrix notation, E × *R* = P, where E, *R,* and P are matrixes containing enrichments, rates, and infusion rates, respectively.

(7) $$\left[\begin{array}{rrrrrr} 1 & 0 & -1 & 1 & -1 & 0\\ 0 & 1 & 1 & -1 & 0 & -1\\ 0 & 0 & -A & B & -A & 0\\ 0 & 0 & C & -D & 0 & -D\\ 0 & 0 & A & -B & 0 & -B\\ 0 & 0 & -C & D & -C & 0 \end{array}\right]\times \left[\begin{array}{c} Ra_{AcAc}\\ Ra_{BHB}\\ Ri_{AcAc}\\ Ri_{BHB}\\ Rd_{AcAc}\\ Rd_{BHB} \end{array}\right]=\left[\begin{array}{c} -Rinf_{AcAc}\\ -Rinf_{BHB}\\ -Rinf_{AcAc}\\ -Rinf_{BHB}\\ 0\\ 0 \end{array}\right]$$

*R* = E^−1^ × P. *R* was solved in Matlab using standard functions.

### 4.9. Metabolic Network and Flux Modeling Using MFA

MFA was performed using a modified version of the INCA software [56], which is based on the elementary metabolite unit (EMU) method [33]. The MFA model is similar in form to the matrix implementation of the two-pool model but incorporates the conversion of AcAc into deuterated BHB during the sample preparation phase (Figure 3A). Atom transitions were generated for the two-pool model, including both carbon and hydrogen atoms (Table 1). An additional reaction capturing the in situ reduction of AcAc to BHB was added to the model. This allowed us to use a complete ketone MID (detected as BHB) containing both BHB and AcAc labeling patterns in the MFA model. Therefore, unlike the matrix method, the MFA model can simultaneously regress all tracer infusion rates, ketone enrichments, and unlabeled fractions of ketones. The MFA model of ketogenesis uses 3 input variables: BHB tracer infusion rate (*Rinf BHB*), AcAc tracer infusion rate (*Rinf AcAc*), and the mass isotopomer distribution of BHB that contains both BHB and AcAc labeling patterns.

### 4.10. Calculation of Precision Score

Precision score was calculated as proposed by Metallo et al. [44]. Briefly, a normalized range is calculated for each flux using the formula:

(8) $$r_{i}=\underset{}{\min}\left(\frac{u_{i}}{\left|v_{i}\right|},\frac{v_{i}}{\left|v_{i}\right|}+1\right)-\underset{}{\max}\left(\frac{l_{i}}{\left|v_{i}\right|},\frac{v_{i}}{\left|v_{i}\right|}-1\right)$$

where *v_i_*, *l_i_*, *u_i_*, and *r_i_* are the estimated flux, lower boundary, upper boundary, and normalized range for the *i*th flux. The individual flux ranges are converted into scores using a negative exponential function:

(9) $$S_{i}=\underset{}{\exp}\left(-\frac{r_{i}}{3}\right)$$

The individual score will have values between 0 and 1. Larger values of *S_i_* indicate narrower confidence intervals and thus more precise estimation of flux.

## 5. Conclusions

In the current study, we investigated technical aspects of a dual-tracer method for estimating in vivo ketone metabolism. Loss of AcAc tracer can occur during neutralization of infusate, but once prepared at neutral pH, the AcAc tracer was stable throughout the infusion procedure. The in situ reduction of AcAc to [^2^H]BHB using NaB^2^H_4_ was rapid and complete, and therefore the resulting BHB MID provided an accurate measure of ketone isotopologues. Analysis of these data in a two-pool model of ketone metabolism constructed in an EMU framework was able to identify problematic variables using residual analysis, quantify the precision of flux estimates, and identify inconsistencies between experimental and modeled data. Using this approach, we observed relationships between individual ketone metabolic fluxes and pools, including the increased interconversion of AcAc to BHB at elevated plasma ketone concentrations. Finally, we discussed the limitations and uncertainty of the individual variables of the model.

## Figures and Tables

**Table 1:** Atom transitions used in the MFA implementation of a two-pool model of ketogenesis. Atom transitions are denoted by letters: capital for carbon atoms and lower case for protons.

Reaction	Atom Transitions
Rates of tracer infusion	
*Rinf BHB*	BHB.inf (AaBCD) -> BHB.blood (AaBCD)
*Rinf AcAc*	AcAc.inf (ABCD) -> AcAc.blood (ABCD)
Rates of appearance	
*Ra BHB*	BHB.source (AaBCD) -> BHB.blood (AaBCD)
*Ra AcAc*	AcAc.source (ABCD) -> AcAc.blood (ABCD)
Rates of interconversion	
*Ri BHB*	BHB.blood (AaBCD) -> AcAc.blood (ABCD) + H.bhb (a)
*Ri AcAc*	AcAc.blood (ABCD) + H.h2o (a) -> BHB.blood (AaBCD)
Rates of disposal	
*Rd BHB*	BHB.blood (AaBCD) -> BHB.tissue (AaBCD)
*Rd AcAc*	AcAc.blood (ABCD) -> AcAc.tissue (ABCD)
Sampling	
*Rs BHB*	0*BHB.blood (AaBCD) -> BHB.s (AaBCD)
*Rs AcAc*	0*AcAc.blood (ABCD) + D.h2o (a) -> BHB.s (AaBCD)
*Sink*	BHB.s (AaBCD) -> SINK

## Data Availability

Original data are available from the authors on request.

## Supplementary Materials

The following are available online at https://www.mdpi.com/article/10.3390/metabo11050279/s1, Figure S1: Real-time reaction monitoring using ^1^H NMR, Figure S2: Comparison of measured and simulated [^2^H]BHB MID, Figure S3: Characteristics of mice used for experiments.
