# Supplementary material for: In Vivo Estimation of Ketogenesis Using Metabolic Flux Analysis—Technical Aspects and Model Interpretation
Source: Metabolites. 2021 Apr 28;11(5):279. doi: 10.3390/metabo11050279 (PMC8146959; doi:10.3390/metabo11050279)
Supplement: Supplementary file 1 [file metabolites-11-00279-s001.zip › metabolites-1165516-supplementary.pdf]

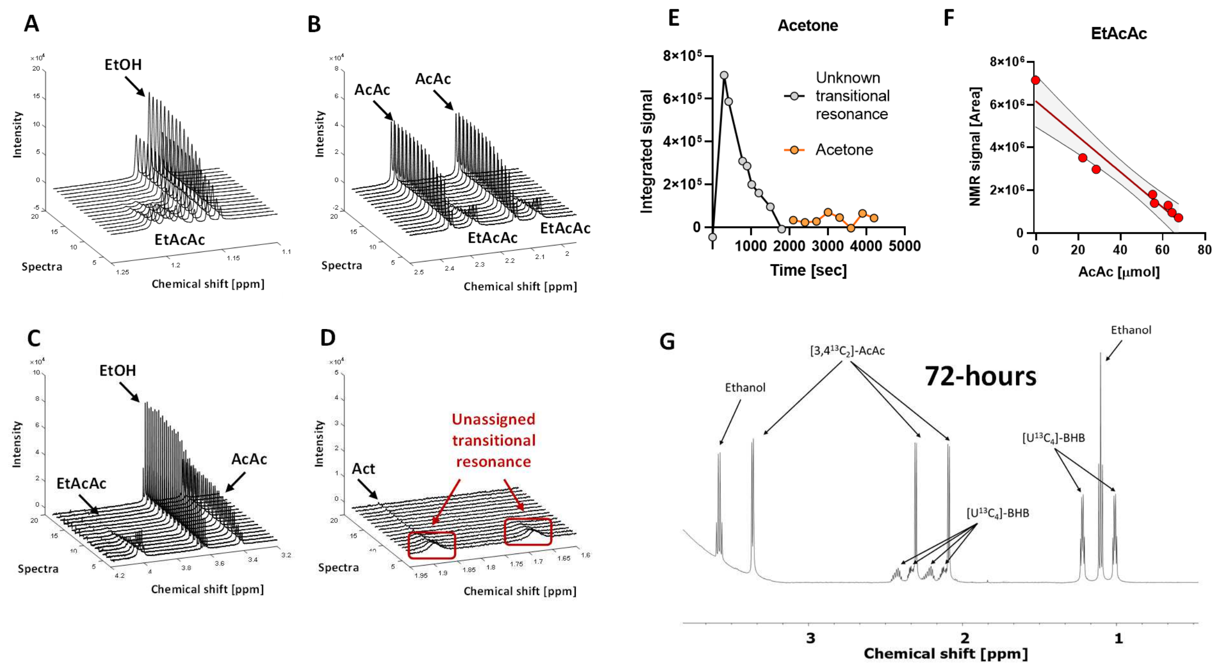

Figure S1. Real time reaction monitoring of EtAcAc hydrolysis to AcAc using ^{1}H NMR. (A-D) Zoomed spectral regions showing signal evolution during real-time reaction monitoring. (E) Integrated ^{1}H NMR resonances of unknown transitional resonance and Acetone over time; (F) relationship between EtAcAc signal detected using ^{1}H NMR and AcAc concentration quantified using enzymatic assay; (G) ^{1}H NMR spectrum of ketone tracer infusate sample after 72-hour storage at room temperature. Both AcAc and BHB resonances are clearly detectable, indicating sufficient stability of AcAc tracer for *in vivo* tracer infusion experiments.

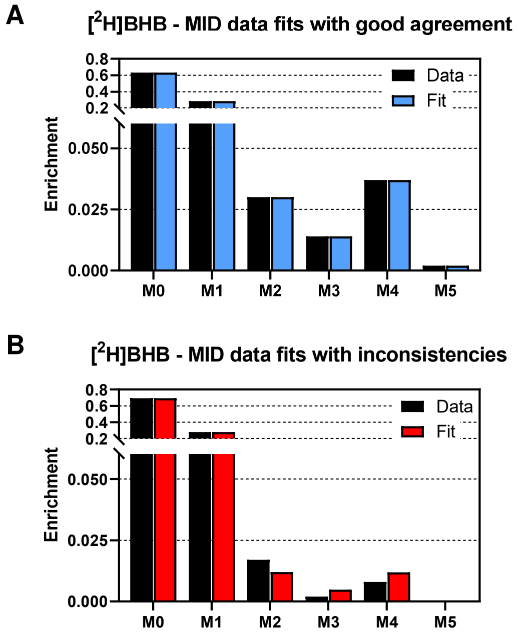

Figure S2. Comparison of measured and simulated [^{2}H]BHB MID. (A) Simulated MID in good agreement with measured values. (B) Simulated MID in disagreement with measurements due to very low enrichment values.

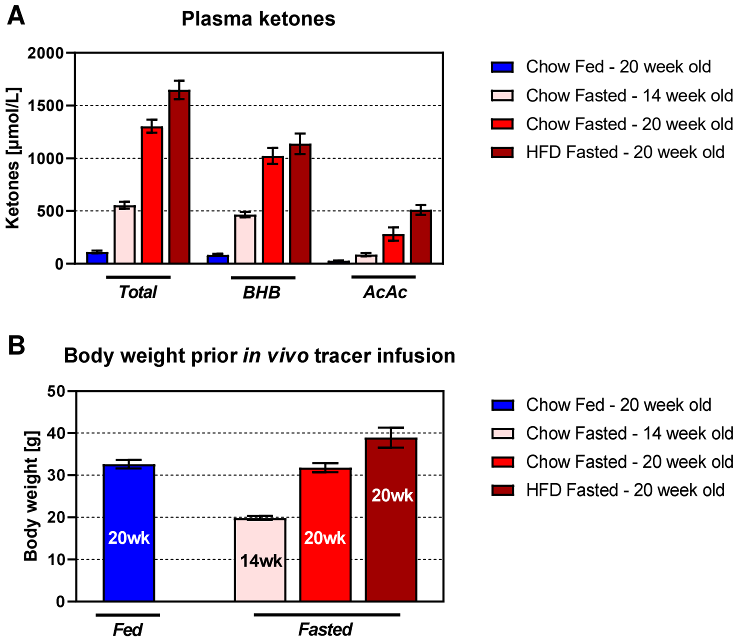

Figure S3. Characteristics of mice used in experiments. (A) Plasma ketones quantified in subset of samples. (B) Body weight prior to *in vivo* tracer infusions.
